# Atomic Imaging of Phase Transformation and Self-Intercalation of Two-Dimensional CrS_2_ by *In Situ* TEM

**DOI:** 10.1021/acsami.5c15768

**Published:** 2025-10-15

**Authors:** Pin-Yu Chou, Hsin-Ya Sung, Che-Hung Wang, Chun-Wei Huang, Wen-Wei Wu

**Affiliations:** † Department of Materials Science and Engineering, 34914 National Yang Ming Chiao Tung University, Hsinchu 30010, Taiwan; ‡ Department of Materials Science and Engineering, 34902 Feng Chia University, Taichung 407, Taiwan; § Center for the Intelligent Semiconductor Nano-system Technology Research, Hsinchu 30078, Taiwan

**Keywords:** high-resolution TEM/STEM, *in situ* heating, self-intercalation, chromium disulfide, layered 2D materials, structural transformation

## Abstract

Controlling phase transitions in two-dimensional (2D) materials offers a powerful route for engineering novel electronic and magnetic functionalities. However, atomically resolved visualization of these dynamic processes remains a significant challenge. Herein, we report the synthesis of ultrathin (1.6 nm), single-crystal 1T-CrS_2_ nanosheets via atmospheric-pressure chemical vapor deposition (APCVD) and uncover their thermal transformation pathway using *in situ* heating transmission electron microscopy (TEM). Real-time atomic-scale imaging reveals that upon heating to 500 °C, the material undergoes an irreversible structural and magnetic transformation from a layered, antiferromagnetic 1T-CrS_2_ structure into a nonlayered, ferrimagnetic Cr_2_S_3_ structure. The driving mechanism is identified as a unique self-intercalation process initiated by the thermal depletion of S atoms, which promotes the migration of lattice Cr atoms into van der Waals (vdW) gaps to form new interlayer covalent bonds. This transformation represents a fundamental dimensional crossover from a 2D vdW crystal to a three-dimensionally bonded material at the nanoscale. Our findings elucidate a critical thermal transformation pathway in Cr-based 2D magnets and demonstrate a mechanism for irreversibly switching both the crystal structure and the magnetic order, thereby providing crucial insights for the design of thermally stable phase-change memory devices. a reduction in the slope of the curve was observed compared with the lower-voltage regime

## Graphical abstract

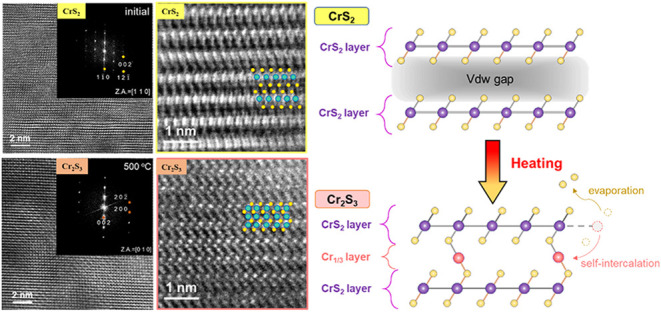

## Introduction

Since graphene was discovered in 2004,^1^ the field of 2D materials, including transition metal oxides, hexagonal boron nitride, transition-metal dichalcogenides (TMDs), has grown explosively. These materials have enriched the diversity of the 2D materials family; among them, TMDs materials have attracted considerable attention owing to their special characteristics, including their tunable bandgaps and outstanding optical and electrical properties, along with the absence of dangling bonds.^2–4^ These characteristics indicate the potential of TMD materials for applications in optoelectronic devices and novel electronics, such as gas sensors,^5,6^ memory devices,^7,8^ field-effect transistors^9,10^ and photodetectors.^11,12^

TMD materials exhibit diverse structures even with the same elemental composition, due to the varying stacking arrangements between the layers, resulting in distinct properties. In these materials, a monolayer consists of a transition-metal atom bonded to a chalcogen atom, both above and below the metal atom, forming a sandwich-like structure. TMDs belong to a family of layered 2D materials in which the layers are held together by vdW forces, giving rise to various crystal structures. The most representative materials are MoS_2_ and WS_2_, which can exist in four distinct structures: 1T, 1T′, 2H, and 3R,^13–16^ with unique physical properties. Furthermore, TMDs can be transformed into different structures by external energy, e.g., annealing to transform 2H-MoS_2_ into 1T-MoS_2_ ^17^ and controlling the synthesis temperature of CrTe_2_ to induce the phase transition to Cr_2_Te_3_ via self-intercalation.^18^

Among TMD materials, Cr-based TMDs with a chemical formula of CrX_2_ (X = S, Se, Te) and their intercalation with nonlayered 2D materials such as Cr_2_S_3_, Cr_2_Te_3_, and CrSe^19–21^ have garnered considerable research interest because of their diverse magnetic behaviors, particularly ferromagnetic behavior. For instance, 1T-CrTe_2_ and CrSe_2_ exhibit ferromagnetic behavior,^22–24^ and according to theoretical calculations,1T, 1T′, 2H-CrS_2_ and Cr_2_S_3_ in a single layer are antiferromagnetic, ferromagnetic, nonmagnetic, and ferrimagnetic, respectively.^25–27^ Furthermore, for 1T-CrS_2_, the thickness effects the magnetic behavior; multilayer 1T-CrS_2_ is ferromagnetic rather than antiferromagnetic and remains ferromagnetic at room temperature.^28^ In addition to their various magnetic properties, CrSe_2_ and 1T-CrS_2_ ^24,28^ exhibit outstanding stability in ambient environments. Owing to these characteristics, Cr-based TMDs have considerable potential for applications in spintronic devices and magnetoresistive random-access memory.^25,26^

As TMDs can exist in multiple phases with the same stoichiometric ratio, TEM and scanning transmission electron microscopy (STEM) are useful techniques for phase identification, providing atomic-resolution structural images along with diffraction information. In addition, the rapid development of *in situ* TEM/STEM observation technique has paved the way for studying atomic motion with subnanometer resolution,^29–35^ which yields direct evidence for phase transitions under different environmental conditions (e.g., annealing and bias). Furthermore, real-time observation by applying heat or bias simulates the conditions of device working environments, providing valuable insights into the engineering design of 2D material-based devices.

Moreover, previous studies on the structural changes and degradation of Cr-based TMDs show the simulation result and Gibbs free energy analysis, reveal the self-intercalation module of Cr-based TMDs.^36^ However, direct evidence for the structural transformation of Cr-based TMDs is still lacking. To enable the future application of Cr-based devices, it is essential to understand how these materials undergo phase transitions under applied energy and to clarify the underlying mechanisms. In this study, we investigated atomic motion during the phase transition of 1T-CrS_2_ by combining *in situ* heating STEM images and high-resolution TEM (HRTEM) images.

## Results and Discussion

In this study, we synthesized high-quality 1T-CrS_2_ using a facile APCVD method, utilizing fluorphlogopite mica as the synthetic substrate because of its similar lattice constant to 1T-CrS_2_.^25,28^ A schematic of the mica synthesis process and a structural diagram of 1T-CrS_2_ are shown in Figure S1a,b, respectively. After synthesis, we performed qualitative and quantitative analyses on the as-grown CrS_2_, as shown in Figure S1c–f, including optical microscopy (OM), scanning electron microscopy (SEM), atomic force microscopy (AFM), and Raman spectroscopy. Comparison with the literature revealed similar thicknesses and Raman peaks (*E*_g_ = 253 cm^–1^, *A*_g_ = 287 cm^–1^).^37,38^ To optimize the APCVD synthesis parameters for obtaining higher-quality CrS_2_ flakes, we systematically recorded the synthesis results to determine the optimal conditions for subsequent research. Schematics of the synthetic method and the setting of precursors are shown in Figure S2a. We also examined the effects of different parameters on the synthesis using optical images, as shown in Figures S2b–i and S3. Owing to the complex interplay of thermodynamics, kinetics, and chemical reactions in chemical vapor deposition (CVD) synthesis, various parameters of the synthesis process, such as the amount of metal precursor, carrier-gas flow flux, and heating temperature, significantly affect the nucleation density, flake size, and morphology of the as-grown CrS_2_ flakes.^39–41^ SEM and AFM analyses were conducted to gain a deeper understanding of the as-grown CrS_2_ flakes with different morphologies and thicknesses (Figures S4 and S5). As shown in Figure S4, SEM revealed various morphologies, including triangular, truncated triangular, hexagonal, and pyramidal shapes. The differences in morphology are attributed to the different ratios of S and Cr precursors used during synthesis, which led to different growth rates at the edges of S and Cr.^41^ Figure S5 shows AFM images and height profiles of CrS_2_ flakes, assuming the ideal thickness of monolayer 1T-CrS_2_ (0.75 nm);^38,42^ the observed CrS_2_ flakes were classified as two to four layers thick. Furthermore, as shown in Figure S5g,h, the thickness of the step-like edge of the pyramidal CrS_2_ flake varied in multiples of the ideal thickness of monolayer CrS_2_.

After the synthesis of CrS_2_ flakes was confirmed, the synthesized product was transferred onto a Cu grid via the wet transfer method to conduct TEM analysis. Figure 1a shows a low-magnification dark-field TEM image of CrS_2_, and Figure 1b shows an HRTEM image of the red-boxed area in Figure 1a, revealing the hexagonal arrangement of atoms. The inset shows the corresponding fast Fourier transform (FFT) along the [0 0 1] axis, indicating that the as-grown CrS_2_ has a single-crystal structure and 6-fold symmetry. In contrast, the annular dark-field STEM (ADF-STEM) image in Figure 1c shows a clear arrangement of Cr and S atoms. A simulated ADF-STEM image of 1T-CrS_2_ (Figure 1d) was compared with the experimental image, revealing identical atomic arrangements and *d*-spacings (2.91 Å) in the [1 1 0] direction. Energy-dispersive X-ray spectroscopy (EDS) elemental mapping and point analysis were used to measure the elemental distribution and composition of the CrS_2_ flake. The uniform distribution of Cr and S in the as-grown CrS_2_ is shown in Figure 1e, and the results of the EDS point analysis were close to the ideal atomic ratio of CrS_2_ (Figure 1f). Additionally, the atomic contrast intensity profiles along the blue lines in Figure 1c,d are compared in Figure 1g and exhibit similar intensity curves. These results confirm the synthesis of 1T-CrS_2_.

**Figure 1:**
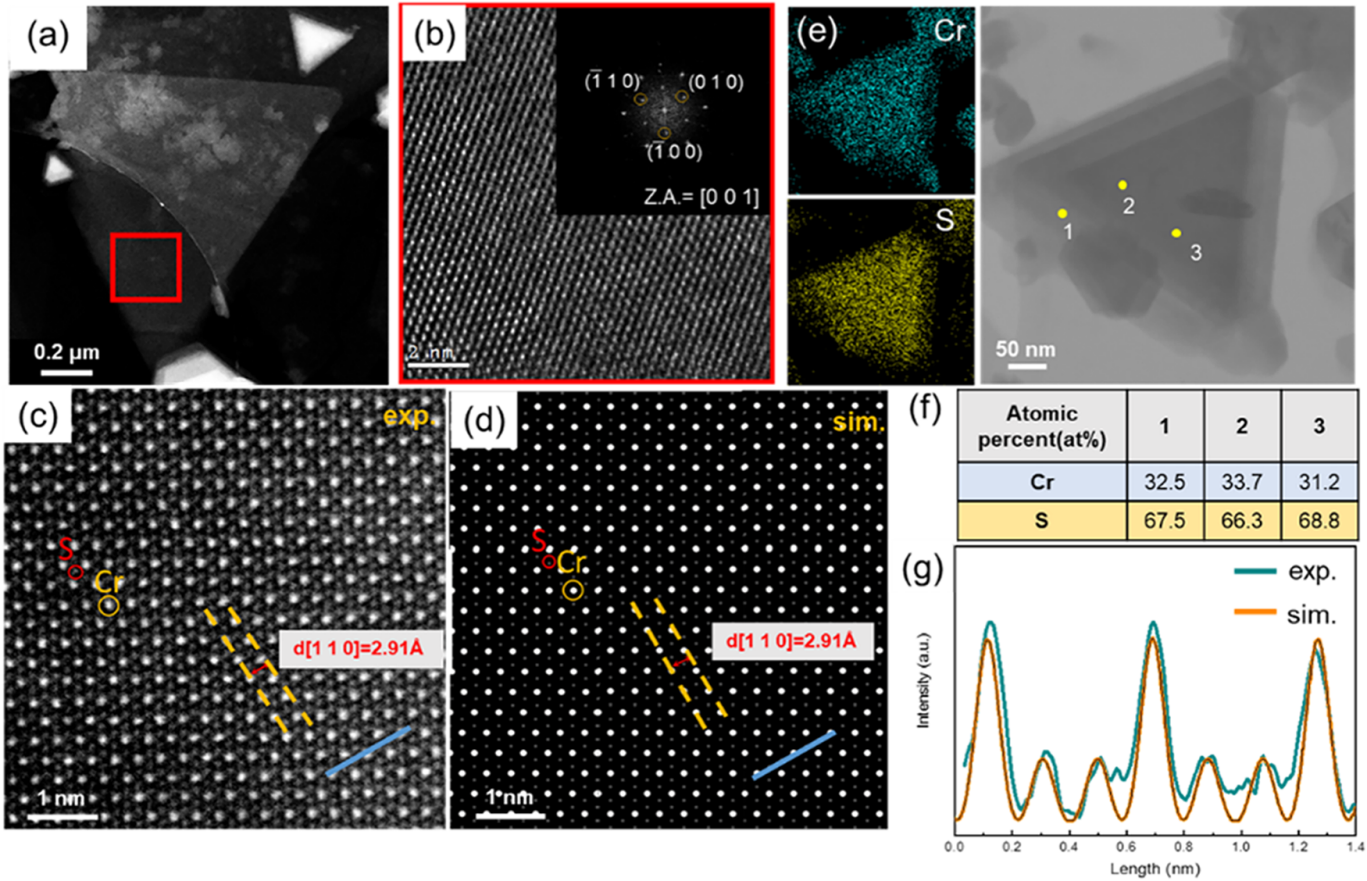
Atomic structure of as-grown 1T-CrS_**2**_. (a) Low-magnification TEM image of CrS_2_. (b) Magnified TEM image of the red-boxed area in (a), with the inset showing the corresponding FFT. (c)-(d) ADF-STEM and simulated STEM images, respectively, indicating the *d*-spacing of CrS_2_. (e) EDS mapping analysis of the low-magnification TEM image. (f) EDS point analysis of the yellow spots in (e). (g) Intensity curves of the blue dashes in (c) and (d).

Except for the TEM image shown in Figure 1, TEM revealed that during the growth of 1T-CrS_2_, small-angle rotations occasionally occurred between the layers. These were more frequently observed in the pyramidal flakes, as shown in Figure S4, because the pyramidal flakes were the results of additional nucleation on the as-grown flakes. In Figure S6, the low-magnification TEM images of 1T-CrS_2_ indicate a slight angular mismatch between the layers, and the HRTEM images with the corresponding FFT display multiple sets of diffraction spots and reveal the moiré fringes (Figure S6d). Figure S7 presents the STEM analysis of the step-like zone in the pyramidal flakes of 1T-CrS_2_, showing clear atomic images of areas of different thicknesses. Both thick and thin areas were captured through focus adjustment, confirming that the atomic arrangements in each region matched those in the simulated STEM image shown in Figure 1d. In addition, the intensity differences between the thick and thin areas were compared using the intensity curve diagrams in Figure S7f. Observing of the boundary between the thick and thin areas, along with the edge between the TEM grid and CrS_2_ (Figure S7e,g), revealed that the as-grown 1T-CrS_2_ had a zigzag edge, which is consistent with previous simulation studies.^43^

After conducting the TEM/STEM structural analysis of 1T-CrS_2_ to clarify the structural changes under external energy, *in situ* TEM techniques were utilized to perform annealing treatment, which allowed real-time observation of the structural transformation in 1T-CrS_2_. After CrS_2_ flakes were transferred onto a special heating chip, they were subjected to *in situ* heating at 500 °C, with exposure to strong electron-beam (e-beam) irradiation. The real-time structural changes in 1T-CrS_2_ are presented in Figure 2. Figure 2a–d show the 1T-CrS_2_ under high-energy e-beam irradiation, with the recording time presented in the bottom-right corner of each image. In Figure 2b, after irradiation for 18 min, hexagonal holes begin to appear on the CrS_2_ surface of the flakes and expand outward gradually. This indicated e-beam-dominated carving behavior, which resulted in sharp, regular hexagonal boundaries on the surface of CrS_2_. According to a previous work,^44^ e-beam carving, which produces regular boundaries, is thermodynamically driven, whereas etching, governed by kinetic factors, tends to result in irregularly shaped holes. As the holes expanded, several merged to form coastline-like boundaries. In Figure 2c, the boundaries of the merged holes can be observed, with the e-beam carving CrS_2_ upward layer-by-layer from the bottom of the image. As shown in Figure 2d, electron beam etching gradually exposed the substrate material on the heated wafer surface, which enhanced the etching effect and further reduced the intensity of the diffraction spots. In addition, clustered regions appeared at the etching boundary, highlighted by yellow circles. Figure 2e presents a magnified view of the area in its initial state, where no significant contrast variation is observed. In contrast, Figure 2f, a magnified view of the yellow-marked region in Figure 2d, reveals a clear contrast difference at the etching boundary, suggesting a change in elemental composition. To verify this, we performed STEM-based elemental analysis of the boundary region. Figure 2g,i present low-magnification ADF-STEM images of the morphology before and after e-beam irradiation, which were used to assess the change in the elemental composition within the e-beam-dominated region. In contrast to Figure 2g, Figure 2i reveals a clear hole for e-beam irradiation, with several clusters observed at the edges of the hole, which is consistent with Figure 2d. EDS analysis revealed that the elemental composition shown in Figure 2h was close to the stoichiometric ratio of CrS_2_, whereas Figure 2j indicates that the clusters are composed of Cr. This result is consistent with previous studies on MoS_2_ and WS_2_ ^45,46^ where pure transition-metal clusters were formed at the edges of the TMD flakes after e-beam irradiation at high temperatures. This phenomenon occurs because of the high energy of the e-beam, which causes significant S atom depletion. After depletion, the Cr atoms in the structure tended to aggregate at the edges to minimize the overall energy because of the higher free energy at the edges.

**Figure 2:**
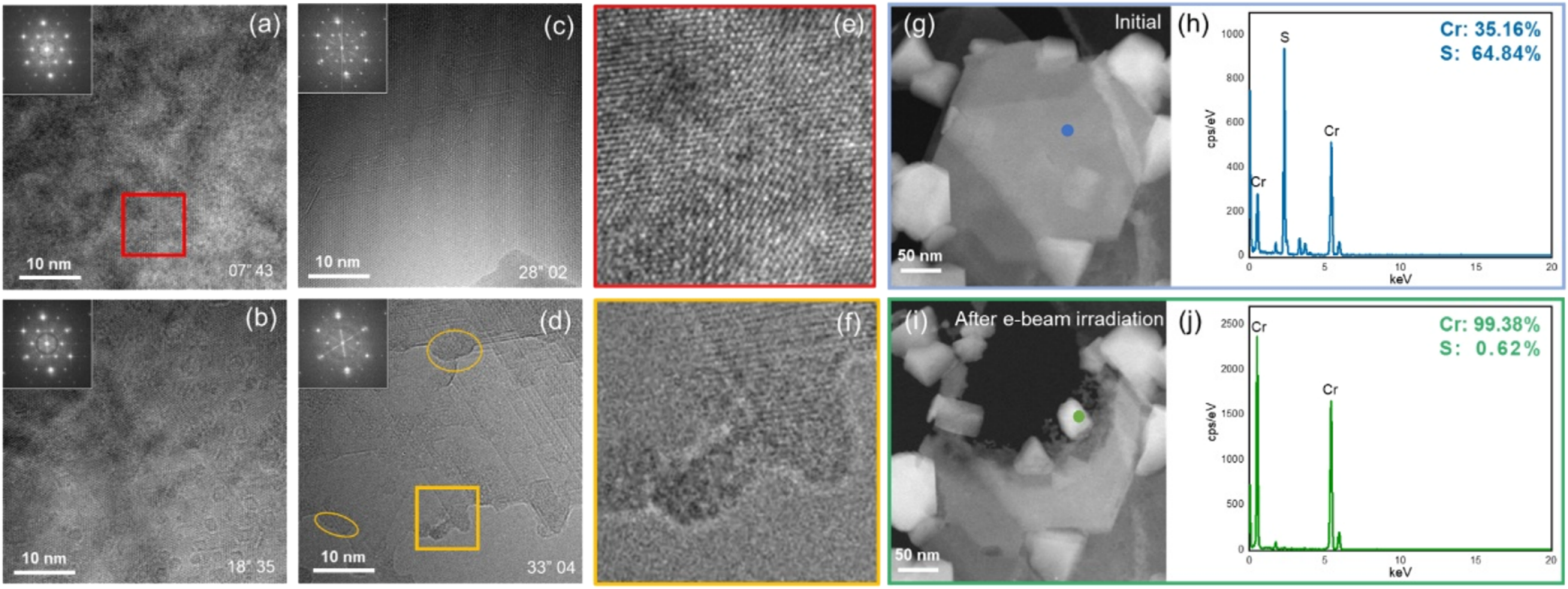
Plan-view TEM images showing the structural transformation sequence of 1T-CrS_**2**_ under high-energy e-beam irradiation. (a)–(d) *In situ* HRTEM images of CrS_2_ under e-beam irradiation over time. (e) High-magnification HRTEM image from the red-boxed region in (a). (f) High-magnification HRTEM image from the yellow-boxed region in (d), showing the Cr cluster area. (g)–(h) Low-magnification ADF-STEM images and EDS analysis of the initial state. (i) Low- magnification ADF-STEM images of the sample after e-beam irradiation damage. (j) EDS analysis of the Cr cluster that emerged at the edge of the damage zone.

A different discovery was made in the region dominated by heating, which was far from the e-beam-dominated zone. As shown in Figure S8, EDS analysis of this area revealed a slight increase in the Cr ratio, with the Cr:S ratio increasing from 1:2 (33.33 at% Cr: 66.66 at% S) to nearly 2:3 (40.00 at% Cr: 60.00% at% S). We deduced that the layered 1T-CrS_2_ might undergo a phase transition to nonlayered Cr_2_S_3_ upon heating. However, observing the structural changes from a cross-sectional angle is essential, because 1T-CrS_2_ and Cr_2_S_3_ exhibit almost the same structures along the [0 0 1] direction. Cross-sectional samples were prepared using a focused ion beam (FIB). A 100 nm-thick SiO_2_ layer was deposited as a protective layer via evaporation before the FIB process to facilitate the subsequent structural analysis. The preparation methods and the structures of the samples are shown in Figure S9.

By applying *ex-situ* and *in situ* annealing to cross-sectional CrS_2_, we examined whether the structural transition of 1T-CrS_2_ was affected by external factors such as pressure and atmosphere. In the *ex situ* annealing, 1T-CrS_2_ was transferred onto a Si substrate and annealed at 600 °C for 2 h under a 200-sccm Ar gas flow as a protective gas at atmospheric pressure. After the annealing treatment, we qualitatively analyzed the sample via Raman spectroscopy (Figure S10) and discovered that the positions of the *E*_g_ and *A*_g_ peaks were slightly shifted. Figure 3a shows a low-magnification cross-sectional TEM image of the initial state of 1T-CrS_2_ with relatively thick flakes (∼20 nm) to facilitate cross-sectional imaging. Figure 3b,c show an HRTEM image and the corresponding FFT diffraction pattern observed along the [1 0 0] direction, which was captured from the yellow-boxed area in Figure 3a. Figure 3d presents a schematic of the atomic structure of 1T-CrS_2_.

**Figure 3:**
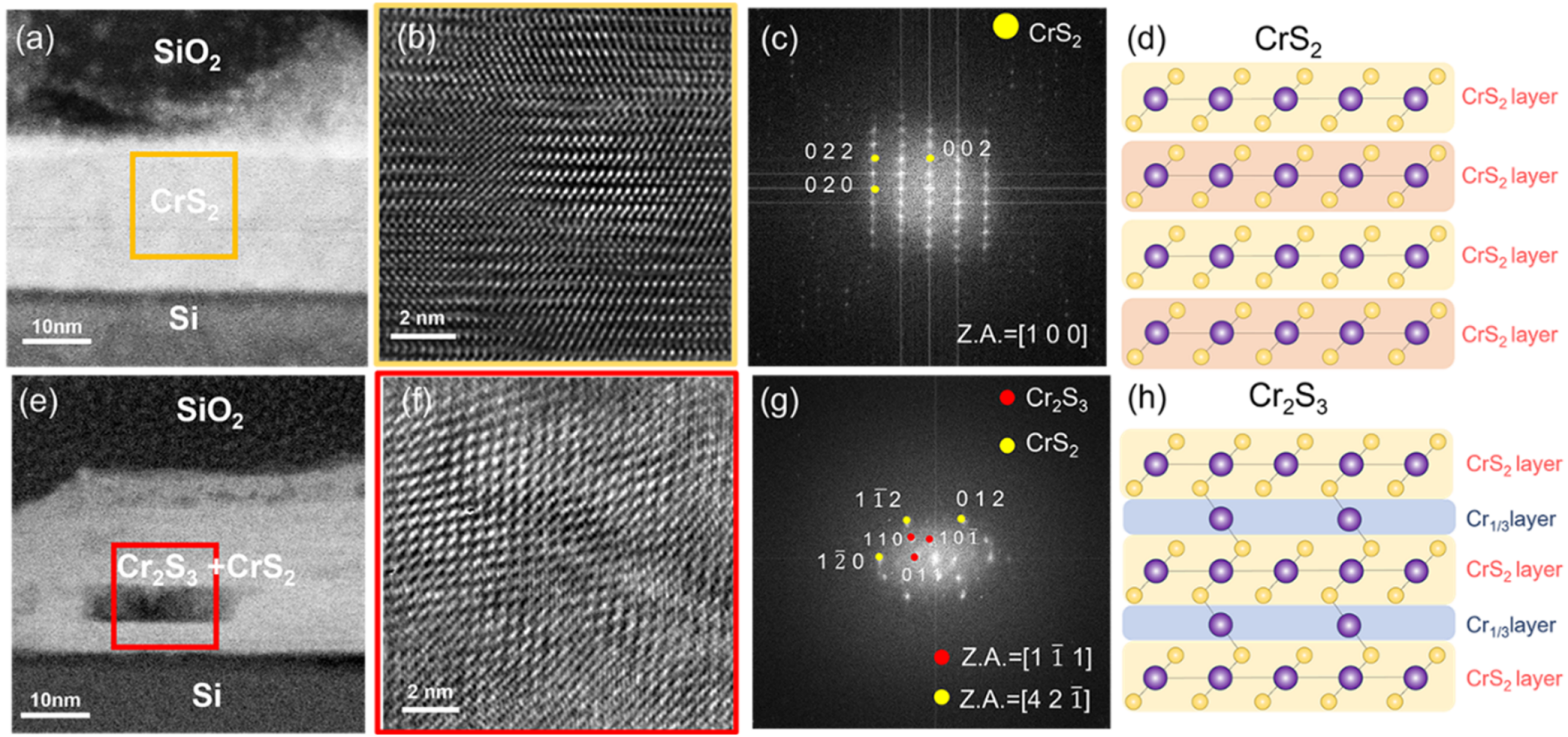
Cross-sectional TEM images showing the phase transformation of 1T-CrS_**2**_ after annealing at 600 °C for 2 h. (a) Initial low-magnification cross-sectional TEM image. (b) Initial HRTEM image and (c) corresponding FFT of CrS_2_. (d) Illustration of the CrS_2_ crystal structure, composed of CrS_2_ layers connected by vdW forces. (e) Low-magnification cross-sectional TEM image after annealing. (f) HRTEM image and (g) corresponding FFT of CrS_2_ after annealing. (h) Illustration of the Cr_2_S_3_ crystal structure, composed of CrS_2_ and Cr_1/3_ layers connected by covalent bonds.

Figure 3e–h present TEM images of 1T-CrS_2_ after *ex situ* annealing, including a low-magnification cross-sectional TEM image, an HRTEM image, the corresponding FFT diffraction pattern, and a schematic of the atomic structure of Cr_2_S_3_, respectively. The atomic arrangement after annealing (Figure 3f) was more disordered than the initial arrangement (Figure 3b). The FFT diffraction pattern in Figure 3g shows two sets of diffraction spots: one set corresponds to 1T-CrS_2_, and the other corresponds to Cr_2_S_3_. The CrS_2_ spots are shown in yellow, whereas the Cr_2_S_3_ spots are shown in red, revealing that the atomic arrangement in Figure 3f is a mixture of 1T-CrS_2_ and Cr_2_S_3_. This indicates a transitional phase from the transition of 1T-CrS_2_ to Cr_2_S_3_. Furthermore, the EDS analysis results before and after annealing are shown in Figure S10, which confirm that the Cr:S ratio increased from approximately 1:2 to 2:3. The EDS results were consistent with the changes in the elemental ratio observed in the heating-dominated region in Figure S8 during plane-view *in situ* annealing. The preliminary results support our hypothesis that the layered 1T-CrS_2_ phase transforms into nonlayered Cr_2_S_3_ in a high-temperature environment. In addition, the atomic structure of Cr_2_S_3_ differs from that of 1T-CrS_2_ (Figure 3d,h); 1T-CrS_2_ consists of repeated CrS_2_ layers connected by vdW forces, forming vdW gaps between the CrS_2_ layers. In contrast, Cr_2_S_3_ features Cr atoms intercalated between the CrS_2_ layers, forming a Cr_1/3_ layer that connects the upper and lower CrS_2_ layers via covalent bonds, which results in a more compact atomic arrangement compared to 1T-CrS_2_. In the following stage, we employed STEM analysis to obtain high-resolution atomic images that revealed the structural distinctions between CrS_2_ and Cr_2_S_3_.

We fabricated CrS_2_-based electronic devices using Ti­(20 nm)/Au­(70 nm) electrodes across a selected CrS_2_ flake. We measured the current–voltage (I–V) curves under different applied-energy conditions. We analyzed the trends of the I–V curves to evaluate the potential structural changes in 1T-CrS_2_ under applied energy. A schematic of the device structure and measurement results are shown in Figure 4. Figure 4d presents the I–V characteristics, which reveal a voltage-dependent trend in the slope of the curve by the same device undergo four voltage ranges from 0 V to 3, 5, 7, 10 V. At lower applied voltages (3 and 5 V), the current value under same voltage shows similar. However, as the voltage increased to higher values (7 and 10 V), the measured current was observed a reduction compared with of lower-voltage regime under same voltage value. This reduction in the slope at elevated voltages suggests an increase in resistance, which is attributed to phase transformations occurring at higher energy levels. Further investigation of the I–V curves measured at different temperatures within the fixed voltage range of −5 to 5 V revealed significant temperature dependence. Specifically, a >10-fold difference was observed between current measurements taken at 200 and 300 °C. This substantial variation highlights the strong influence of the temperature on the electrical conductivity of the material. The 1T-CrS_2_ device exhibited the most significant changes in the measurement results at 300 °C. The current values measured at 300 °C were an order of magnitude higher than those measured at 200 °C. This considerable variation is attributed to temperature-induced structural recovery to enhance crystallinity or phase changes occurring at 300 °C. *In situ* heating experiments were conducted to clarify these findings.

**Figure 4:**
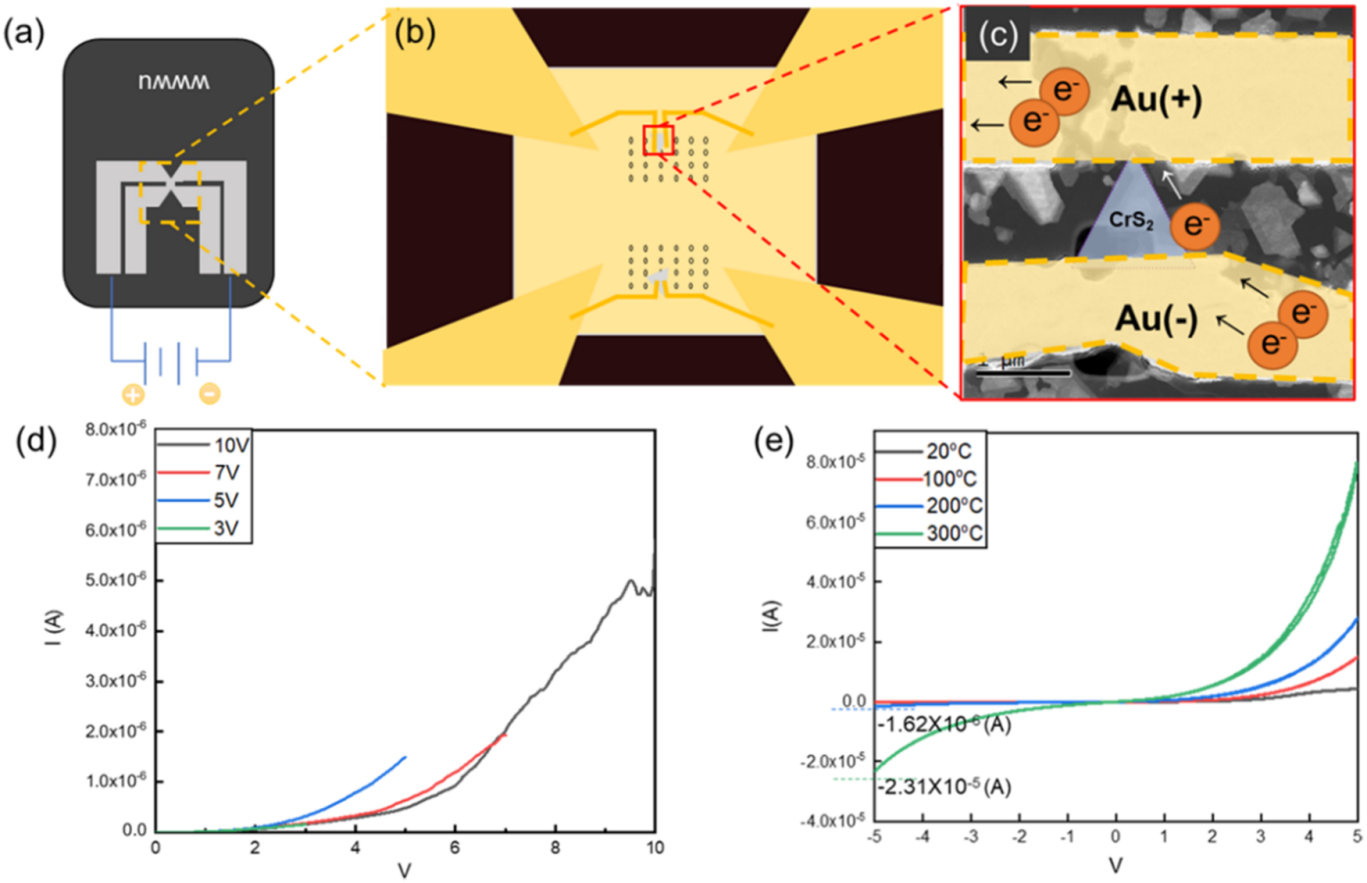
Preparation and electrical analysis of the CrS_**2**_-based device. (a) Schematic of the electrical chip. (b) CrS_2_-based device configuration set on the observation window of the electrical chip. (c) Low-magnification TEM image of the device. (d) I–V curve acquired with the voltage changing from 3 to 10 V. (e) I–V curve acquired with the temperature increasing from 20 to 300 °C.

In the *in situ* heating experiments, the intense etching effects of the high-energy e-beams were avoided. The e-beam was turned off during the heating process to minimize damage. After the sample was held at the target temperature for a specified duration, the e-beam was reactivated to capture TEM/STEM images. The experiment commenced at 300 °C, and the temperature was increased in 100 °C increments. At each step, the temperature was maintained for 15 min, and HRTEM images were captured at the end of each step until the temperature reached 600 °C. Figure 5a shows a cross-sectional STEM image of 1T-CrS_2_ before heating, with Cr and S atoms marked by blue and yellow dots, respectively. The atomic arrangement shown in Figure 5a is consistent with the schematic of the 1T-CrS_2_ atomic structure shown in Figure 3d, and the presence of clear vdW gaps between the CrS_2_ layers is a characteristic of layered materials. In contrast, the STEM image captured after *in situ* heating (Figure 5b) showed a noticeable change in the resulting structure corresponding to the atomic arrangement of Cr_2_S_3_ as illustrated in Figure 3h, where the atoms are tightly bonded and the vdW gap disappears. We utilized HRTEM and the corresponding FFT images to further characterize the material after the phase transition. Figure 5c,d show the HRTEM and corresponding FFT images in the initial state and after heating to 300 °C, respectively. The analysis indicated that the material in both cases was 1T-CrS_2_ along the [1 1 0] direction. However, at 300 °C, a significant increase in the intensity of the FFT diffraction spots was observed. This is attributed to the recovery that occurred within the material as the temperature increased, which improved the crystallinity. Accordingly, the aforementioned increase in current at elevated temperatures (Figure 4) is attributed to the enhanced crystallinity. When the temperature was increased to 500 °C (Figure 5e), the diffraction pattern changed, revealing Cr_2_S_3_ along the [0 1 0] direction. Additionally, the HRTEM images in Figure 5e show a more close-packed atomic arrangement, consistent with the aforementioned phase transition from 1T-CrS_2_ to Cr_2_S_3_. With an increase in the temperature to 600 °C, the FFT image in Figure 5f reveals two additional rows of diffraction spots compared to Figure 5e, which were identified as Cr_3_S_4_ and Cr_5_S_6_. This result is consistent with a previous study^34^ on *in situ* heating of Cr_2_S_3_, indicating that at temperatures between 500 and 600 °C, self-intercalation effects lead to the phase transition of Cr_2_S_3_ to Cr_3_S_4_ and Cr_5_S_6_. However, these phases existed only at high temperatures and reverted to Cr_2_S_3_ upon cooling to room temperature. This confirmed that the material after the thermally induced phase transition was Cr_2_S_3_, demonstrating that *in situ* heating and *ex situ* annealing produce the same result and are unaffected by external environmental conditions. Hence, the present study provides an effective method for converting 1T-CrS_2_ into Cr_2_S_3_.

**Figure 5:**
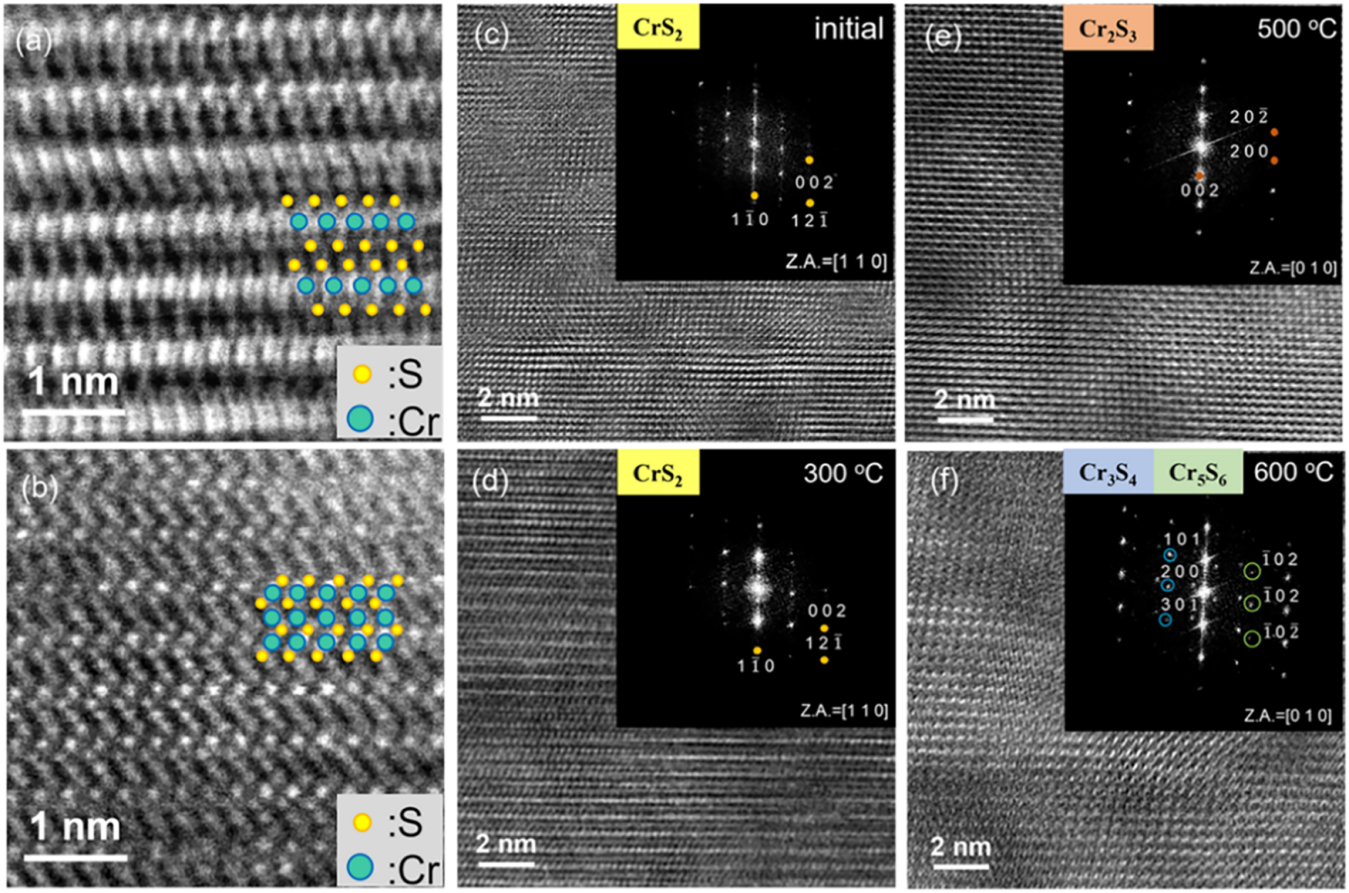
In-situ cross-sectional STEM/TEM images showing the phase transformation of 1T-CrS_**2**_ at different temperatures for 15 min. (a) ADF-STEM image of 1T-CrS_2_ in the initial state, where green and yellow spots represent Cr and S atoms, respectively. (b) ADF-STEM image of Cr_2_S_3_ at room temperature after *in situ* heating, showing the structural transformation of CrS_2_. (c) HRTEM and corresponding FFT images of the initial state of CrS_2_ in the [1 1 0] direction. (d) HRTEM and corresponding FFT images at 300 °C, with a higher intensity of FFT diffraction spots. (e) HRTEM and corresponding FFT images at 500 °C, showing Cr_2_S_3_ in the [0 1 0] direction. (f) HRTEM and corresponding FFT images at 600 °C, showing Cr_3_S_4_ and Cr_5_S_6_ in the [0 1 0] direction, as indicated by blue and green circles, respectively.

Our findings confirmed that 1T-CrS_2_ can be transformed into Cr_2_S_3_ via a simple annealing treatment, and the following discussion focuses on the mechanism underlying this transformation. Figure 6 presents a schematic of the phase transformation of 1T-CrS_2_, as depicted in Figure 2, which shows that exposure to a high-energy e-beam causes the depletion of S atoms in CrS_2_, leading to the formation of pure Cr clusters. Previous research^45^ indicates that compared to transition metals, S atoms, owing to their lower threshold knock-on energy, are more prone to reaching their critical energy when subjected to external energy, resulting in bond breakage and ejection from the CrS_2_ structure. Although the energy applied during heating is not as intense per unit area as that of a high-energy e-beam, the heating process can cause S atoms to break bonds in a high-temperature environment and disperse from the structure without inducing significant damage. As indicated by the EDS results, the atomic percentage of S was reduced after heating, decreasing from 66.6% to 60.0%. The depletion of S atoms by heating generated vacancies, increasing the defect concentration within the 1T-CrS_2_ crystal. The depletion effect of the S defect concentration increased, which increased the free energy of the Cr atoms near the defects, promoting bond breakage and the subsequent self-intercalation of Cr atoms into the CrS_2_–CrS_2_ interlayers to stabilize the structure. This self-intercalation process resulted in the formation of Cr_1/3_ layers between the CrS_2_ layers, transforming the crystal structure from a layered 1T-CrS_2_ (CrS_2_–CrS_2_) configuration, where the layers were weakly connected by vdW forces. In contrast, the nonlayered Cr_2_S_3_ (CrS_2_–Cr_1/3_-CrS_2_) is interconnected through strong chemical bonding, indicating robust interlayer interactions compared to typical vdW-layered materials. A comprehensive schematic of the atomic dynamic movement is provided in Figure 6.

**Figure 6:**
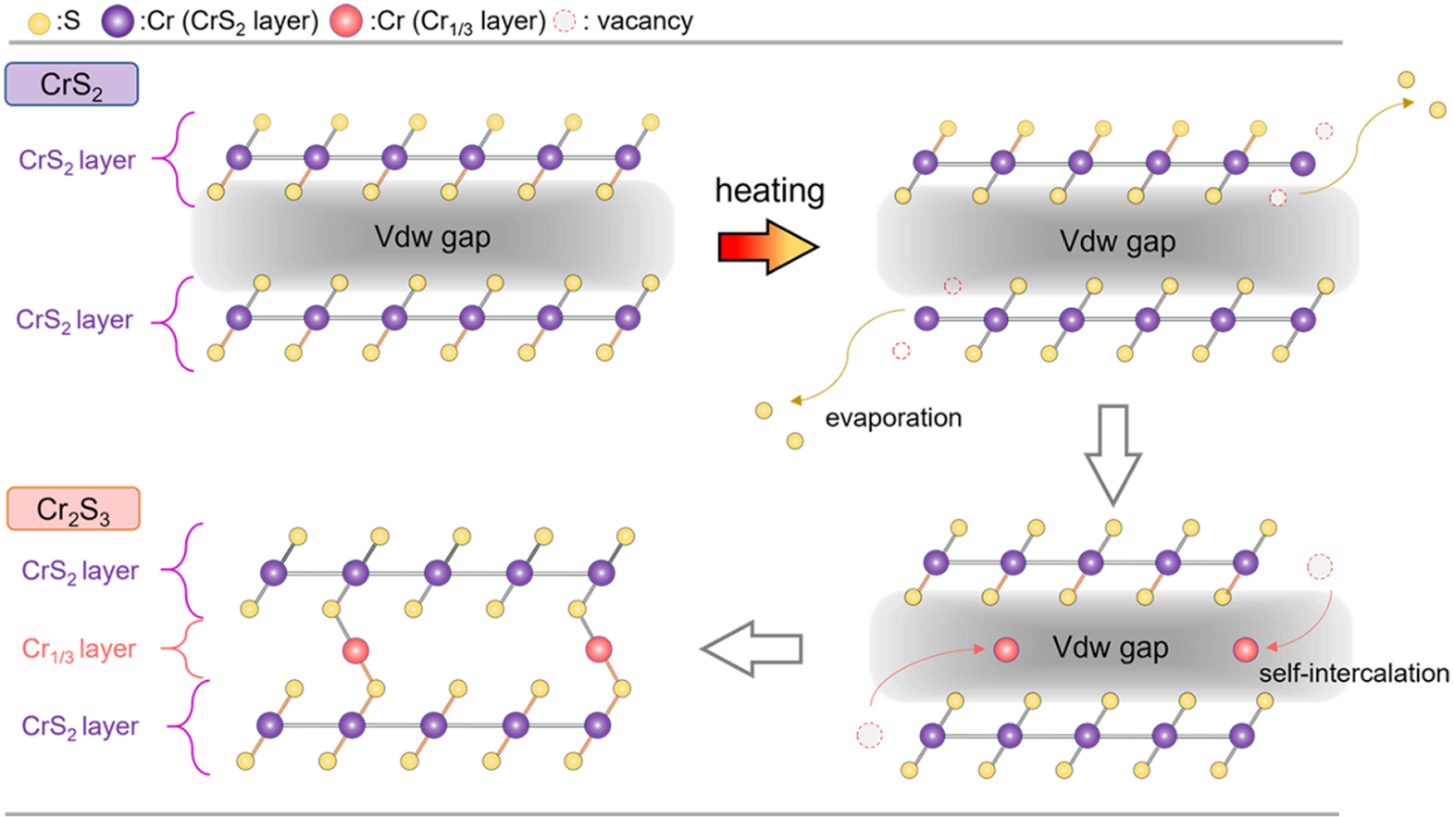
Schematic projection diagram of the phase transformation of 1T-CrS_2_ to Cr_2_S_3_ through self-intercalation behavior.

## Conclusions

We systematically investigated the thermal stability and transformation pathways of ultrathin, single-crystal 1T-CrS_2_ synthesized via APCVD. Through rigorous *in situ* TEM experiments, we decoupled the effects of e-beam irradiation, which causes localized etching and Cr cluster formation, from the global thermal response. Our primary finding, confirmed by both *ex situ* and *in situ* cross-sectional analysis, was a thermally induced, irreversible phase transformation from a layered, antiferromagnetic 1T-CrS_2_ structure to a nonlayered, ferrimagnetic Cr_2_S_3_ structure at 500 °C. We identified the underlying mechanism as a unique self-intercalation process: the thermal depletion of S atoms increases the Gibbs free energy of the system, driving Cr atoms to migrate from the parent lattice into the vdW gaps. This forms new Cr_1/3_ intercalated layers and replaces weak interlayer forces with strong covalent bonds, representing a fundamental dimensional crossover from a two-dimensionally bonded material to a three-dimensionally bonded material. The present study provides the first atomic-scale visualization of this transformation in a Cr-based TMD and offers critical insights into its thermal stability. Understanding how to induce irreversible structural and magnetic phase changes is vital for designing robust spintronic and phase-change memory devices based on Cr chalcogenides.

## Methods

### APCVD Growth of CrS_2_

A two-zone furnace was used to synthesize CrS_2_ under atmospheric pressure. S powder (99%) was placed in the front heating zone upstream, and Cr powder (99%) mixed with NaCl powder (99.5%) as a supporting agent was positioned in the center heating zone of the furnace for CVD. The temperature of the center heating zone was gradually increased to 765 °C and maintained for 25 min. Mica substrates were placed directly above the precursors. During this process, the Ar flow rate was set at 90 sccm. After completion of the reaction, the furnace was cooled to room temperature.

### Transfer Method for CrS_2_ Samples

A wet-transfer method using poly­(methyl methacrylate) (PMMA) was employed to transfer the as-grown CrS_2_ onto specialized TEM heating chips, TEM Cu grids, or Si substrates. PMMA was spun onto a mica substrate, baked at 130 °C for 1 min, and soaked in an HF solution (20 wt %) for 2 h. The etched PMMA films with CrS_2_ were separated with mica, rinsed with deionized water, and removed using acetone at 70 °C for 3 h.

### Fabrication of CrS_2_ Devices

The CrS_2_ samples were transferred to an electrical chip with Si_3_N_4_ membrane windows via a wet transfer process. Methyl methacrylate (bottom layer) and PMMA (top layer) were spin-coated onto the chip as positive-tone photoresist (PR), followed by baking at 180 °C for 1 min via e-beam lithography. Ti (20 nm) and Au (70 nm) were deposited as electrodes using an electron-gun evaporation system (E-gun). Subsequently, the PR was removed via lift-off using acetone within 24 h. The distance between the two electrodes was 1 μm.

### Microstructural Characterization of Materials

The samples were characterized via OM, SEM, AFM, and Raman spectroscopy to study their morphology, determine the sample thickness, and examine the characteristics of CrS_2_. The structural evolution of CrS_2_ was observed using field-emission TEM (JEOL-F200) at a 200 kV accelerating voltage, and the elemental distribution was analyzed using EDS (Oxford EDS 100 TLE).

### TEM/STEM with Heating Holder

The *in situ* experiments were performed using a special TEM heating system, including a prototype fusion select holder and tilt controller (Audro 300), a power supply (2616A System Sourcemeter), and a software controller (Fusion 350 V1.0.0). DF-STEM images were obtained using TEM with a Cs corrector (JEOL JEM-ARM200FTH) at a 200 kV accelerating voltage.

## Supplementary Material



## Notes

Details of CrS_2_ Synthesis and Characterization: OM, SEM, and AFM analyses of the CrS_2_ samples; TEM, STEM, and EDS images during and after the in situ experiments; and the sample preparation procedure for cross-sectional specimens (PDF)
