# Supplementary material for: Atomic Imaging of Phase Transformation and Self-Intercalation of Two-Dimensional CrS2 by In Situ TEM
Source: ACS Appl Mater Interfaces. 2025 Oct 15;17(43):59665–73. doi: 10.1021/acsami.5c15768 (PMC12581116; doi:10.1021/acsami.5c15768)
Supplement: Supplementary file 1 [file am5c15768_si_001.pdf]

Supporting Information

Atomic Imaging of Phase Transformation and Self-Intercalation of Two-Dimensional CrS_2 by In Situ TEM

*Pin-Yu Chou[1, a], Hsin-Ya Sung[1, a], Che-Hung Wang[1], Chun-Wei Huang[2, *] and Wen-Wei Wu[1, 3, *]*

[1] Department of Materials Science and Engineering, National Yang Ming Chiao Tung University, Hsinchu 30010, Taiwan

[2] Department of Materials Science and Engineering, Feng Chia University, Taichung 407, Taiwan

[3] Center for the Intelligent Semiconductor Nano-system Technology Research, Hsinchu, 30078, Taiwan

[a] Pin-Yu Chou and Hsin-Ya Sung contributed equally.

[*] Correspondence and requests for materials should be addressed to C.-W. H. and W.-W. W (email: huangcw@fcu.edu.tw and wwwu@nycu.edu.tw)

List of contents

Figure S1 | **Synthesis and characteristics of 1T-CrS_2.**

Figure S2 | **Synthetic method and different parameters affecting the growth of CrS_2 flakes.**

Figure S3 | **Optical images showing the effects of different temperatures on CrS_2 flake growth.**

Figure S4 | **SEM images of CrS_2 flakes with different morphologies synthesized via APCVD.**

Figure S5 | **AFM images of CrS_2 flakes with different heights and numbers of layers.**

Figure S6 | **Interlayer rotation of multilayer CrS_2.**

Figure S7 | **ADF-STEM images of the pyramidal CrS_2 flake edge sequence, from thick to thin.**

Figure S8 | **EDS results showing the variation in the atomic ratio of the non-e-beam-dominated zone.**

Figure S9 | **Schematic of cross-sectional CrS_2 sample preparation.**

Figure S10 | **Qualitative anlysis of the *ex situ*-annealed CrS_2 sample (600 °C/2 h).**

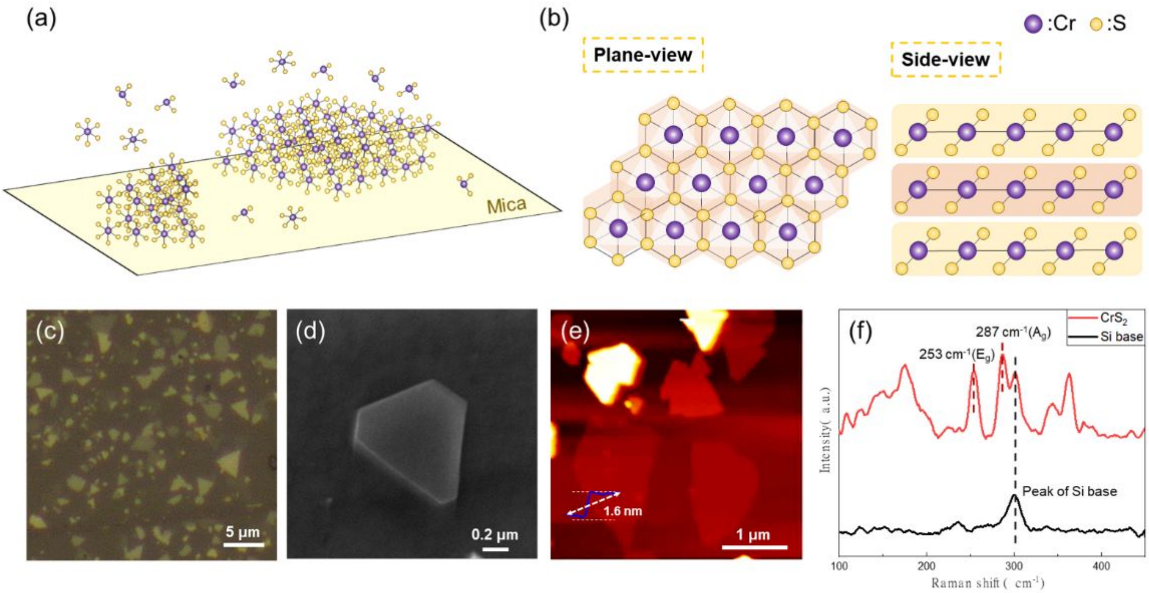

Figure S1. Synthesis and characteristics of 1T-CrS_2.

(a) Illustration of the CrS_2 growth process on mica. (b) Plan-view and side-view structural schematics of CrS_2. (c) Optical image of as-grown CrS_2. (d) SEM image of CrS_2. (e) AFM image and corresponding height profile of a CrS_2 nanosheet. (f) Raman spectra of CrS_2 crystals, with the characteristic peaks of E_g and A_g labeled.

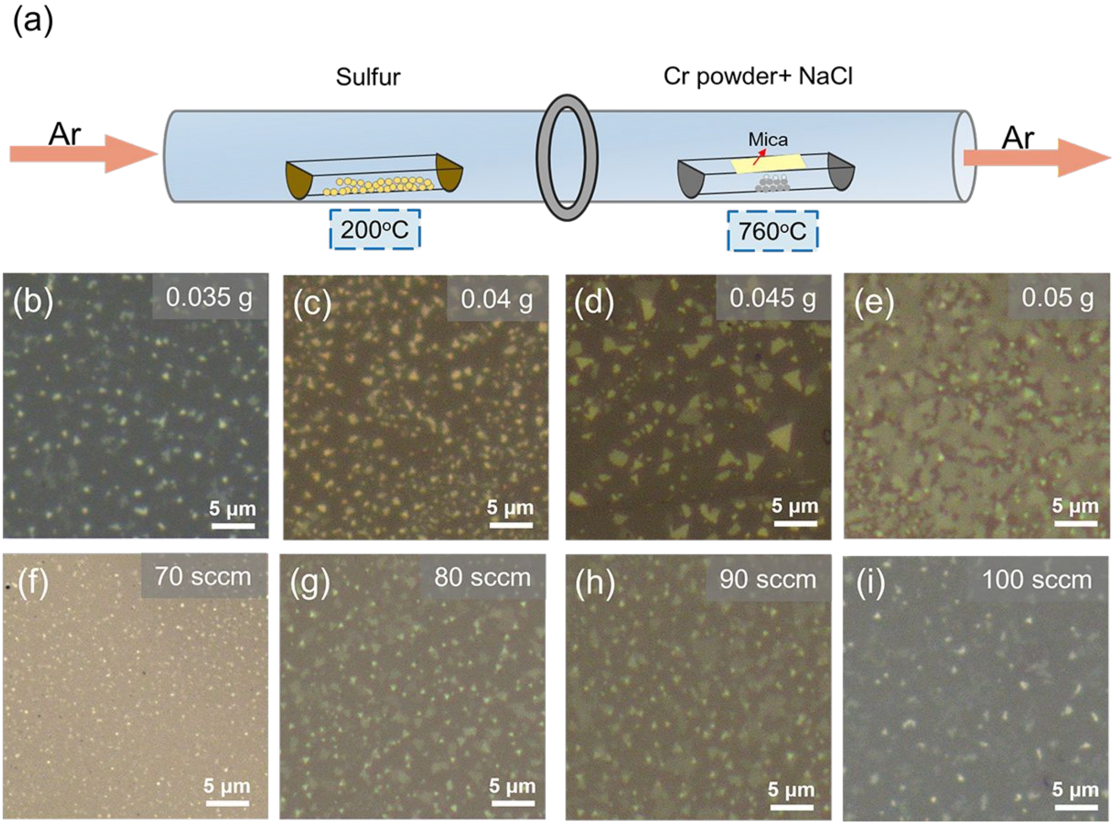

Figure S2. Synthetic method and different parameters affecting the growth of CrS_2 flakes. (a) Schematic of CrS_2 synthesis via APCVD. (b)–(e) Optical images of CrS_2 growth with different amounts of metal precursor: (b) 0.035 g; (c) 0.04 g; (d) 0.045 g; (e) 0.05 g. (f)–(i) Optical images of CrS_2 growth with different Ar fluxes: (f) 70 sccm; (g) 80 sccm; (h) 90 sccm; (i) 100 sccm.

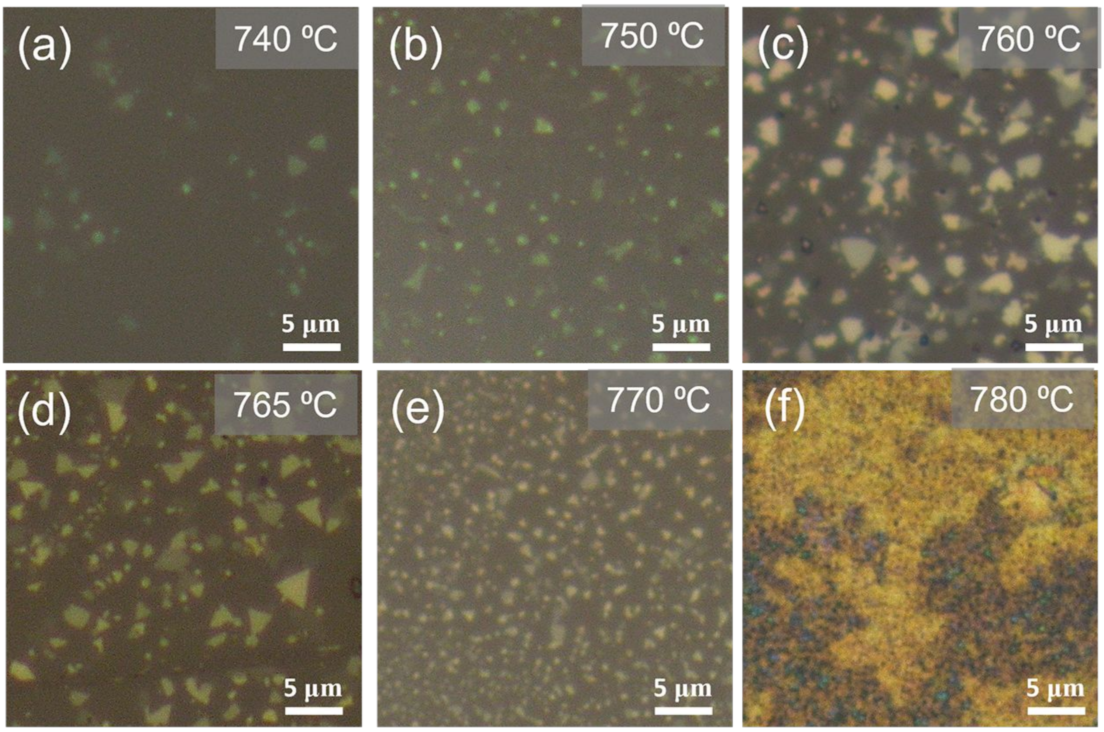

Figure S3. Optical images showing the effects of different temperatures on CrS_2 flake growth.

(a) 740 °C; (b) 750 °C; (c) 760 °C; (d) 765 °C; (e) 770 °C; (f) 780 °C.

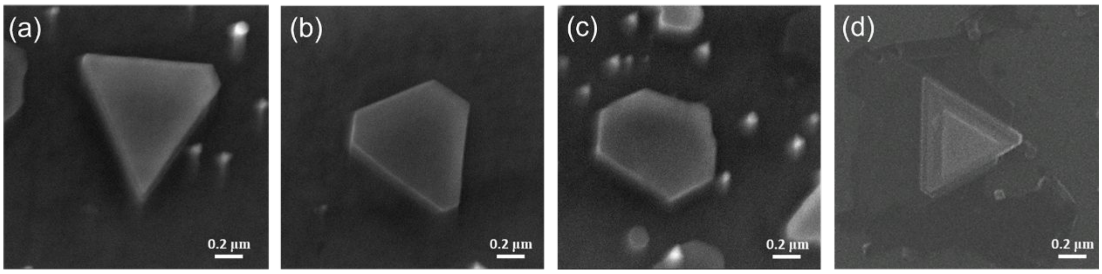

Figure S4. **SEM images of CrS_2 flakes with different morphologies synthesized via APCVD.**

(a) triangular shape; (b) truncated triangular shape; (c) hexagonal shape; (d) pyramidal shape.

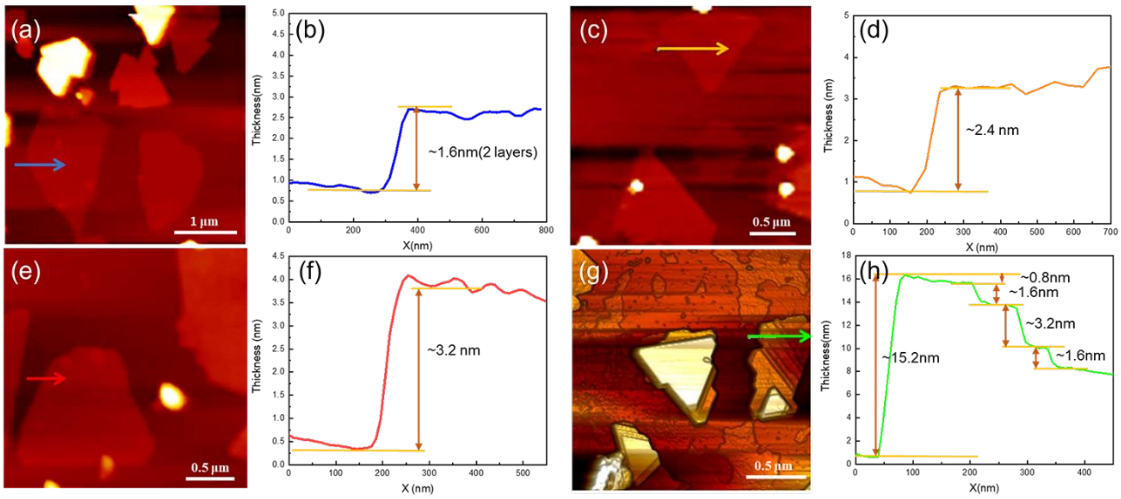

Figure S5. AFM images of CrS_2 flakes with different heights and numbers of layers.

(a)-(b) AFM image of two layers of CrS_2 flakes with the corresponding height profile along the blue arrow. (c)-(d) AFM image of three layers of CrS_2 flakes with the corresponding height profile along the yellow arrow. (e)-(f) AFM image of four layers of CrS_2 flakes with the corresponding height profile along the red arrow. (g)-(h) AFM image of pyramidal CrS_2 flakes with the corresponding height profile along the green arrow, showing multiples of the single-layer CrS_2 height.

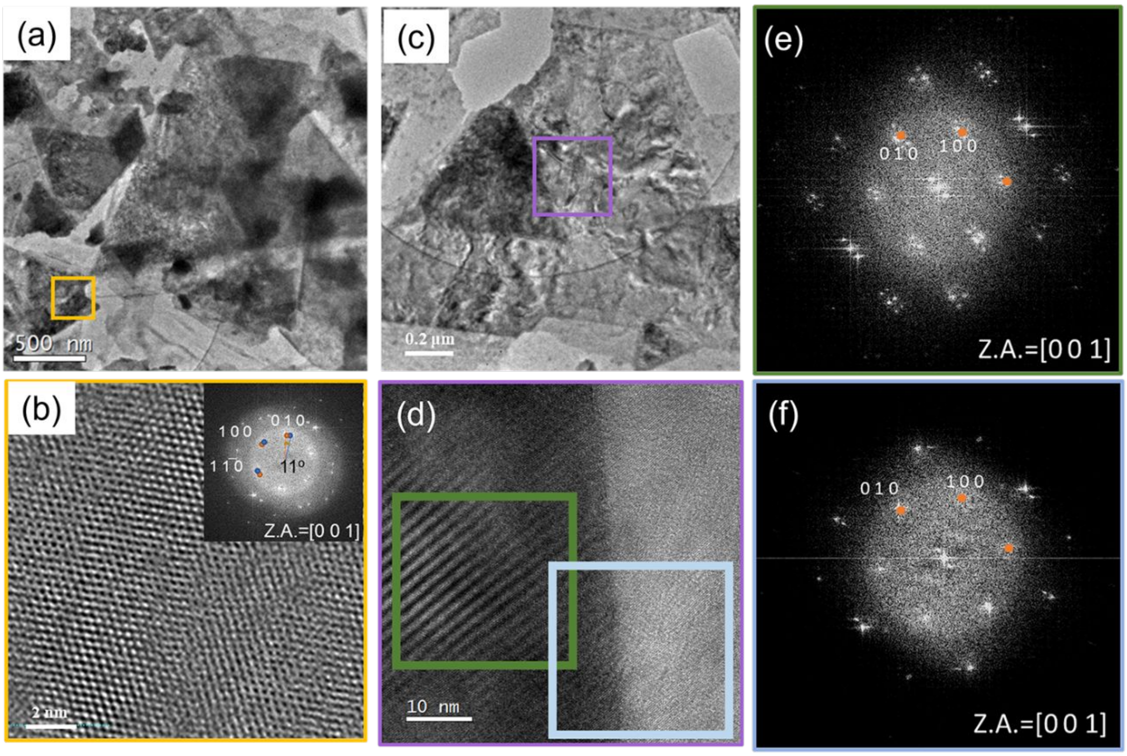

Figure S6. **Interlayer rotation of multilayer CrS_2.**

(a) Low-magnification image of multilayer CrS_2. (b) HRTEM image of the yellow-boxed area in (a); the corresponding FFT inset shows 11 degrees of rotation between CrS_2 layers. (c) Low-magnification image of pyramidal CrS_2. (d) HRTEM image of the purple-boxed area in (c), showing moiré fringe in the flake overlapping zone. (e)-(f) Corresponding FFT of (d) with overlapping (green box) and non-overlapping zones (light blue box) shown in (e) and (f), respectively.

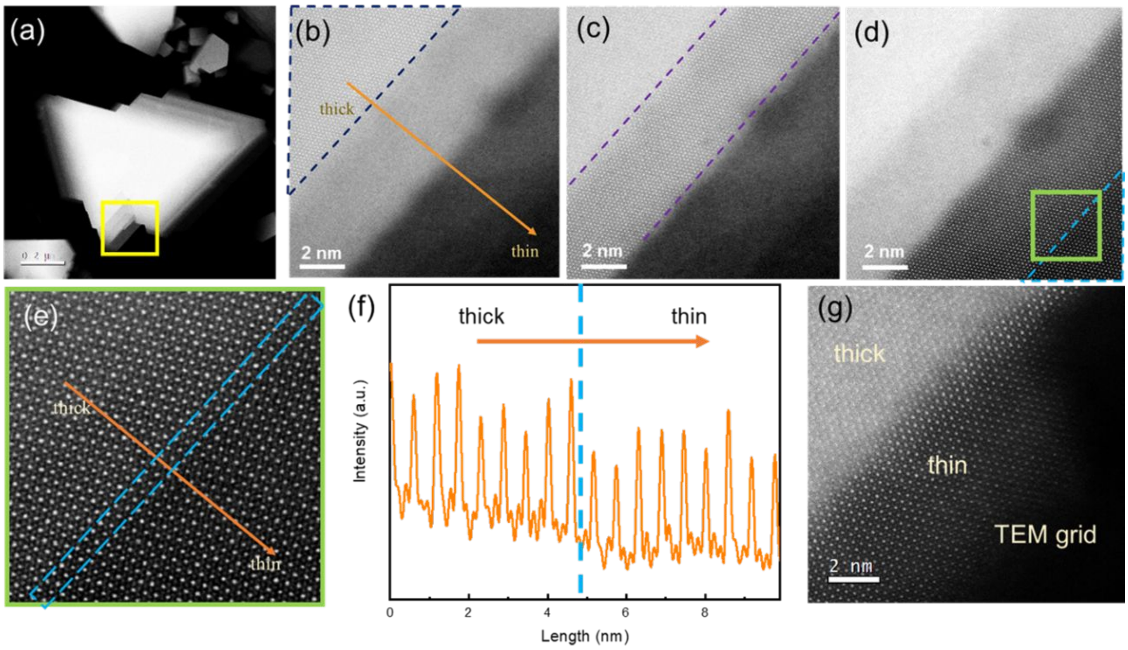

Figure S7. **ADF-STEM images of the pyramidal CrS_2 flake edge sequence, from thick to thin.**

(a) Low-magnification STEM image of pyramidal flake; the subsequent images were photographed in the yellow-boxed area. (b)-(d) High-resolution STEM images of the stair-like area; areas of different thicknesses were photographed by adjusting the focus. (e) Magnified image of the green-boxed area in (d), with the blue dashed line indicating the edge between different thickness zones. (f) Intensity curve along the orange arrow shown in (e), which indicates that the intensity is positively correlated with the thickness. (g) Boundary between the TEM grid and CrS_2, showing the zig-zag edge of CrS_2 flakes.

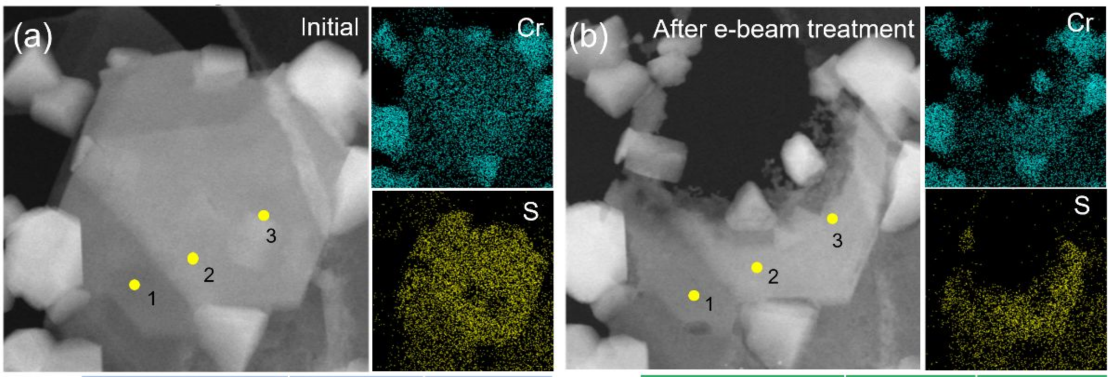

(c)

Spectrum Label(At%)	Cr	S
Spectrum 1	37.10	62.90
Spectrum 2	32.02	67.98
Spectrum 3	35.18	64.82

(d)

Spectrum Label(At%)	Cr	S
Spectrum 1	39.72	60.28
Spectrum 2	38.42	61.58
Spectrum 3	40.84	59.16

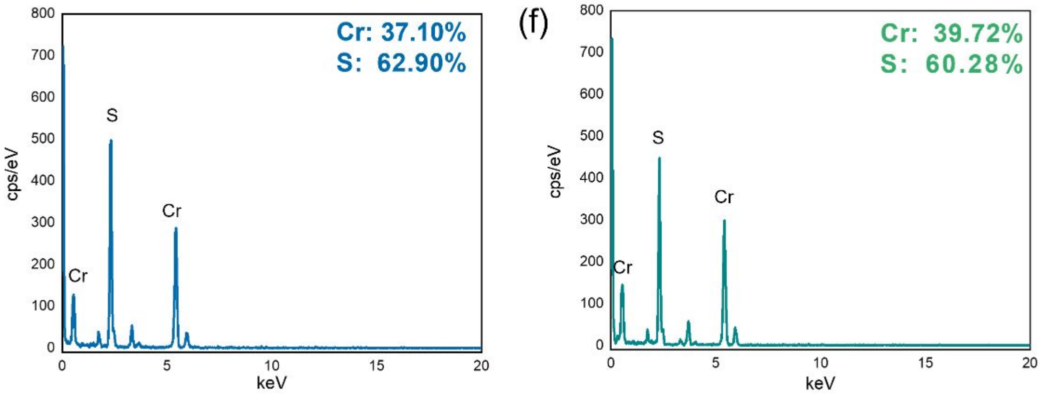

Figure S8. **EDS results showing the variation in the atomic ratio of the non-e-beam-dominated zone.**

(a)-(b) Low-magnification TEM images with EDS mapping analysis before and after e-beam treatment, respectively. (c) EDS point analysis of yellow spots in (a). (d) EDS point analysis of yellow spots in (b), far from the e-beam-damaged area. (e) Intensity diagram corresponding to the EDS point analysis in (a). (f) Intensity diagram corresponding to the EDS point analysis in (c).

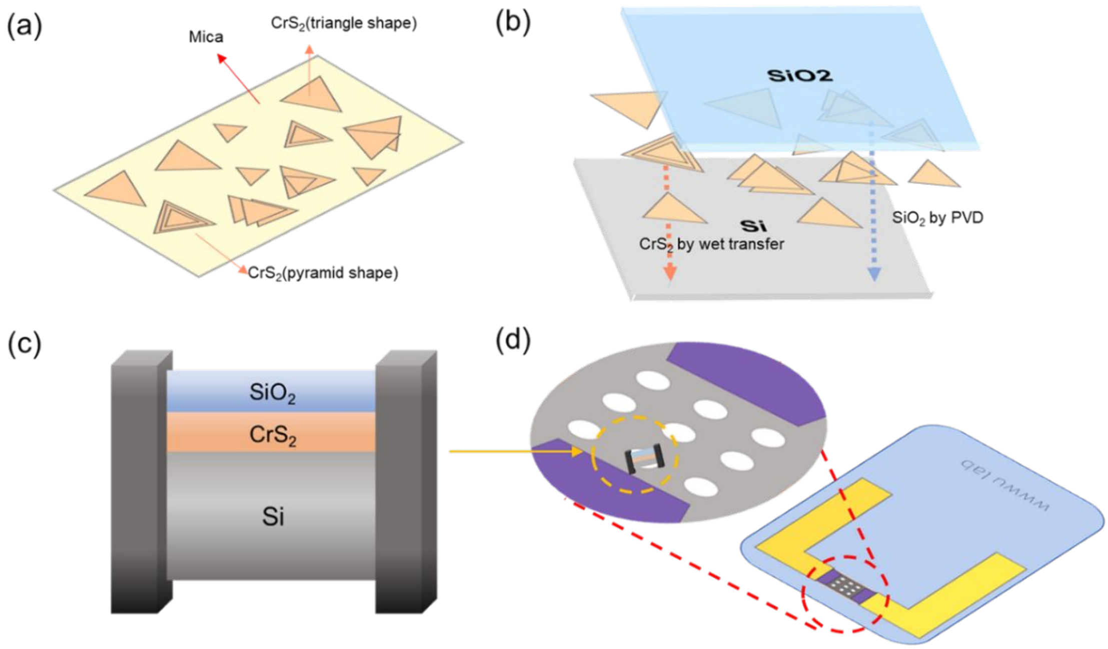

Figure S9. **Schematic of cross-sectional CrS_2 sample preparation.**

(a) Schematic of the mica substrate after APCVD. (b) Schematic of the process before FIB. (c) Illustration of the structure of the FIB-milled cross-sectional sample. (d) Illustration of the cross-sectional sample on the observation window of the *in situ* heating chip.

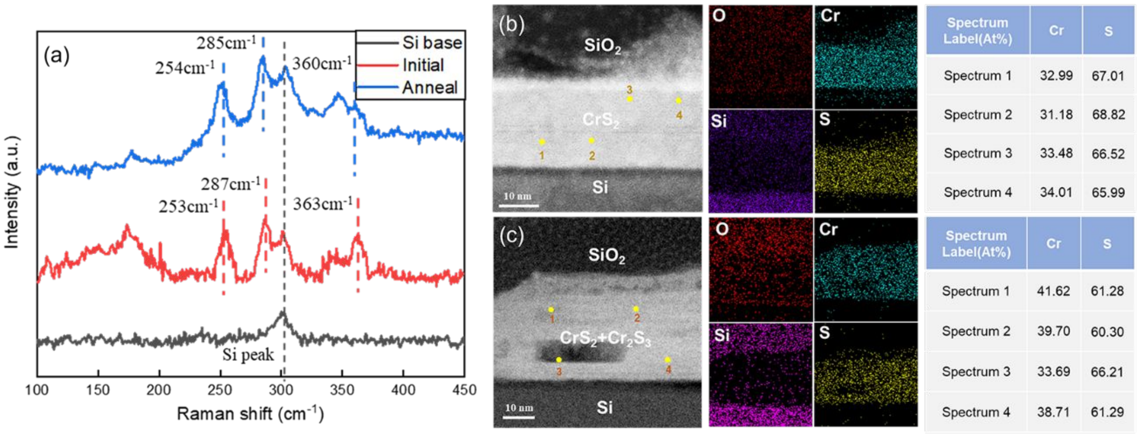

Figure S10. **Qualitative anlysis of the *ex situ* annealed CrS_2 sample (600 °C/2 h).** (a) Raman spectra of the initial and annealed samples. Low-magnification TEM images and EDS analysis of the cross-sectional CrS_2 sample (b) in the initial state and (c) after annealing.
